# Feasibility and acceptability of SARS-CoV-2 testing and surveillance in primary school children in England: Prospective, cross-sectional study

**DOI:** 10.1371/journal.pone.0255517

**Published:** 2021-08-27

**Authors:** Felicity Aiano, Samuel E. I. Jones, Zahin Amin-Chowdhury, Jessica Flood, Ifeanyichukwu Okike, Andrew Brent, Bernadette Brent, Joanne Beckmann, Joanna Garstang, Shazaad Ahmad, Frances Baawuah, Mary E. Ramsay, Shamez N. Ladhani

**Affiliations:** 1 Public Health England, London, United Kingdom; 2 Derbyshire Healthcare NHS Foundation Trust, Derby, United Kingdom; 3 Oxford University Hospitals NHS Foundation Trust, Oxford, United Kingdom; 4 University of Oxford, Oxford, United Kingdom; 5 East London NHS Foundation Trust, London, United Kingdom; 6 Birmingham Community Healthcare NHS Trust, Aston, United Kingdom; 7 Manchester University NHS Foundation Trust, Manchester, United Kingdom; 8 Public Health England, Manchester Royal Infirmary, Manchester, United Kingdom; 9 Paediatric Infectious Diseases Research Group, St. George’s University of London, London, United Kingdom; Freelance Consultant, Myanmar, MYANMAR

## Abstract

**Background:**

The reopening of schools during the COVID-19 pandemic has raised concerns about widespread infection and transmission of SARS-CoV-2 in educational settings. In June 2020, Public Health England (PHE) initiated prospective national surveillance of SARS-CoV-2 in primary schools across England (sKIDs). We used this opportunity to assess the feasibility and agreeability of large-scale surveillance and testing for SARS-CoV-2 infections in school among staff, parents and students.

**Methods:**

Staff and students in 131 primary schools were asked to complete a questionnaire at recruitment and provide weekly nasal swabs for SARS-CoV-2 RT-PCR testing (n = 86) or swabs with blood samples for antibody testing (n = 45) at the beginning and end the summer half-term. In six blood sampling schools, students were asked to complete a pictorial questionnaire before and after their investigations.

**Results:**

In total, 135 children aged 4–7 years (n = 40) or 8–11 years (n = 95) completed the pictorial questionnaire fully or partially. Prior to sampling, oral fluid sampling was the most acceptable test (107/132, 81%) followed by throat swabs (80/134, 59%), nose swabs (77/132, 58%), and blood tests (48/130, 37%). Younger students were more nervous about all tests than older students but, after completing their tests, most children reported a “better than expected” experience with all the investigations. Students were more likely to agree to additional testing for nose swabs (93/113, 82%) and oral fluid (93/114, 82%), followed by throat swabs (85/113, 75%) and blood tests (72/108, 67%). Parents (n = 3,994) and staff (n = 2,580) selected a preference for weekly testing with nose swabs, throat swabs or oral fluid sampling, although staff were more flexible about testing frequency.

**Conclusions:**

Primary school staff and parents were supportive of regular tests for SARS-CoV-2 and selected a preference for weekly testing. Children preferred nose swabs and oral fluids over throat swabs or blood sampling.

## Introduction

The emergence of SARS-CoV-2 and its rapid spread has led to mass disruption globally, with implementation of lockdown measures that have included school closures in most countries. In England, the first imported cases appeared in late January and started to increase rapidly in March 2020 [1]. In response, schools were closed on Friday 20 March 2020 in England, with the exception of vulnerable children and children of key workers [2], who were allowed to attend school throughout the national lockdown [3]. National lockdown, with closures of hospitality venues and non-essential shops followed on 23 March 2020 [4]. Cases continued to increase until mid-April and then declined gradually until the end of the national lockdown on May 2020 [5]. On 01 June 2020, primary schools for pupils aged between 4 and 11 years reopened, starting with reception, school years 1 and 6, until the end of the summer half-term in mid-July 2020 [6]. Schools were advised to implement social distancing measures, ensuring staff and students were limited to “bubbles of 15 students” to minimise contact and facilitate contact tracing of cases, and maintaining two metres distance when possible and appropriate [6].

Typically, children develop asymptomatic or mild illness when exposed to SARS-CoV-2 and rarely require hospitalisation [7]. Their role in transmission however, remains uncertain. Early in the COVID-19 pandemic, studies in Australia and Iceland reported low levels of SARS-CoV-2 infection and transmission in educational settings [8, 9]. Concerns remain about the potential for educational settings to become infection hubs, where children spread the infection both to other children and staff, and also to potentially vulnerable household members and into the wider community [10]. To better understand SARS-CoV-2 infection and transmission in educational settings, Public Health England (PHE) initiated prospective surveillance in primary schools across England in June 2020 (COVID-19 Surveillance in school KIDs, sKIDs) [11]. Around 12,000 staff and students were recruited in 131 primary schools across England within two weeks of schools reopening on 01 June 2020. Staff and students in participating schools provided weekly nasal swabs for the whole of the summer half-term (01 June to mid-July 2020), or provided swabs, oral fluid and blood samples at the beginning and the end of the summer half-term.

Such large-scale testing in this demographic and setting has not been done before. Accordingly, to better understand the feasibility, acceptability and frequency of testing for SARS-CoV-2 in educational settings, we asked primary school staff and parents of primary school students to complete a short questionnaire at recruitment. In six schools where blood samples were taken from participants, we also asked primary school students to complete a short picture questionnaire before and after their SARS-CoV-2 tests. This information is important for developing safe school policy and establishing SARS-CoV-2 testing in educational settings during the pandemic.

## Methods

### sKIDs surveillance

Public Health England (PHE) initiated SARS-CoV-2 surveillance in primary schools in May 2020 [11]. The sKIDs protocol is available online (https://www.gov.uk/guidance/covid-19-paediatric-surveillance). Primary schools across England were contacted to take part in the sKIDs surveillance. Pupils and staff members at participant schools were tested for SARS-CoV-2. The study comprised of two arms, with different sampling regimes;

- Weekly swabbing arm: nose swabs (weekly intervals from the 1^st^ June 2020 in schools with minimum 30 children and open for a minimum of 4 weeks) and one oral fluid sample at end of term.
- Blood sampling arm: blood samples, nose and throat swabs and oral fluid samples (OFS) (three study visits June, July and November 2020).

### Staff and parent questionnaires

At the first sampling session in June 2020, after providing written informed consent, primary school staff completed a questionnaire at recruitment, which included optional questions about their acceptance, opinions, feasibility and frequency of testing for SARS-CoV-2 in school, with space for free text comments (**S1 File**). In primary schools involved with blood sampling, participating staff were also asked about their acceptability and frequency of blood tests to monitor SARS-CoV-2 antibodies (**S1 File**). Parents of primary school children also provide written informed consent and were asked the same questions for their child (**S1 File**). All parents, staff and children were invited to complete the questionnaire which was voluntary, however not all participants completed the questionnaire before and after their tests, and some only completed part of the questionnaire which was included in the analysis.

### Student experiences

Participating primary school students were asked to complete an anonymised closed multi-choice paper questionnaire at one of the sKIDs sites (North London), which included six primary schools (**S2 File**) before and after their tests at the first visit. Both sKIDs investigators and teachers helped each student complete the questionnaire. The first part of the questionnaire was completed prior to any tests being done. Questions included a simple written and pictorial explanation of each type of sample (nose swab, throat swab, blood sample and oral fluid sample) and asked children to rate on a scale how they were feeling about the sample (e.g. “very nervous” to “looking forward to it”). The Wong-Baker pain emoji faces were used to rate any discomfort associated with each sampling method [12]. All children were invited to complete the questionnaire which was voluntary, however not all children completed the questionnaire before and after their tests, and some only completed part of the questionnaire which was included in the analysis. 135 children out of a of 244 from the six schools fully or partially completed the questionnaire. The questionnaire was anonymous and optional for children to complete.

### Swabbing participation

We assessed rates of participation in the swabbing arm of the study by calculating the return percentages of participants from their first week of sampling. Because not all schools joined the study in the same week, the number of available sampling weeks (eligibility) differed between participants, with schools open for between two and six weeks of sampling. Moreover, because participants sometimes joined after the first sampling week at their school, we calculated eligible weeks at the level of the participant. For example, if a participant joined in the second week of sampling at a school that was open for the full six weeks of the study, the participant would have five eligible weeks of participation.

### Data analysis

Data were entered into Microsoft Access and analysed using Stata v.15.0 (Statacorp, Tx). Data are mainly descriptive. Categorical variables are presented as proportions and compared using the chi-squared or Fisher’s Exact test as appropriate. MAXQDA Analytics Pro 2020 (Release 20.2.2) was used for coding of free texts. Only legible comments that were relevant to the question “what is the feasibility and acceptability of COVID-19 testing in schools?” were coded. Data were analysed using an iterative thematic framework approach. Drawing on current knowledge about parental attitudes regarding paediatric phlebotomy, and the research question, initial codes were considered prior to analysis (Parent/guardian comments, staff comments which were then sub-coded as appropriate to nose, throat, oral fluid, blood, or *other*), with scope to develop further codes as appropriate. The frequency of codes was counted in relation to feasibility and acceptability, as a measure of importance, with new codes added as appropriate.

### Ethics approval

The sKIDs study was approved by the Public Health England Research Ethics Governance Group (R&D REGG Ref: NR0209, 16 May 2020).

## Results

In total, 131 schools were recruited into the sKIDs primary school surveillance. Of these, 86 schools comprising 9,592 participants (5761 students, 3232 staff, 599 unidentified) were involved in weekly swabbing and 45 schools comprising 2427 participants (994 students, 1420 staff, 13 unidentified) provided a nose swab, throat swab, oral fluid and a blood sample at the beginning and at the end of the summer half-term. The median age of children in the study was 8 years old (IQR: 6–10 years, range: 4–12 years).

### Children’s questionnaire on acceptability of testing

Children’s experiences were assessed in six London schools participating in the blood sampling arm of sKIDs. A total of 134 children aged 4–7 years (n = 40) or 8–11 years (n = 95) completed partially or fully completed the questionnaire about their experience with nasal swabs, throat swabs, oral fluid and blood tests before and after sampling.

#### Before testing

Prior to the tests, oral fluid sampling (107/132, 81%) was reported by the children to be the most acceptable test, compared to the throat swabs (80/134, 59%), nose swabs (77/132, 58%), and blood tests (48/130, 37%) (**Table 1**). The students were most concerned about the blood test, with nearly two-thirds reporting feeling “very nervous” (41/130, 32%) or “a little nervous” (41/130, 32%). Prior to testing, students aged 4–7 years were more likely to feel nervous about the nose swab (26/39 [67%] vs. 29/93 [31%]; P = 0.020) compared to 8–11 year-olds. Both age groups were similarly nervous about blood tests (24/38 [63%] vs. 58/92 [63%]; P = 0.995) (**Table 1**).

**Table 1:** Children’s reported expectations before the test, and experiences after the test.

	4–7 year-olds (n = 39)				8–11 year-olds (n = 95)				All children pre-test
	*Expectations before test*	*Experience after the test*			*Expectations before test*	*Experience after the test*			*Expectations before test*
**NOSE SWAB**		*Better*	*As expected*	*Worse*		*Better*	*As expected*	*Worse*	
Very nervous	7/39 (18%)	4/4 (100%)	0/4 (0%)	0/4 (0%)	10/93 (11%)	6/9 (67%)	2/9 (22%)	1/9 (11%)	**17/132 (13%)**
A little bit nervous	19/39 (49%)	5/14 (36%)	5/14 (36%)	4/14 (28%)	19/93 (20%)	10/17 (59%)	7/17 (41%)	0/17 (0%)	**38/132 (29%)**
Don’t mind, not worried	5/39 (13%)	4/5 (80%)	1/5 (20%)	0/5 (0%)	48/93 (52%)	19/42 (45%)	22/42 (53%)	1/42 (2%)	**53/132 (40%)**
Looking forward to it	8/39 (20%)	6/8 (75%)	2/8 (25%)	0/8 (0%)	16/93 (17%)	5/12 (42%)	7/12 (58%)	0/12 (0%)	**24/132 (18%)**
		**19/31 (61%)**	**8/31 (26%)**	**4/31 (13%)**		**40/80 (50%)**	**38/80 (47%)**	**2/80 (3%)**	
**THROAT SWAB**									
Very nervous	9/39 (23%)	3/7 (43%)	2/7 (28.5%)	2/7 (28.5%)	8/95 (8%)	4/7 (57%)	2/7 (29%)	1/7 (14%)	**17/134 (13%)**
A little bit nervous	10/39 (26%)	5/9 (56%)	2/9 (22%)	2/9 (22%)	27/95 (28%)	9/24 (38%)	14/24 (58%)	1/24 (4%)	**37/134 (28%)**
Don’t mind, not worried	14/39 (36%)	3/9 (33%)	5/9 (56%)	1/9 (11%)	51/95 (54%)	16/44 (36%)	22/44 (50%)	6/44 (14%)	**65/134 (48%)**
Looking forward to it	6/39 (15%)	4/5 (80%)	1/5 (20%)	0/5 (0%)	9/95 (10%)	6/8 (75%)	1/8 (12%)	1/8 (13%)	**15/134 (11%)**
		**15/30 (50%)**	**10/30 (33%)**	**5/30 (17%)**		**35/83 (42%)**	**39/83 (47%)**	**9/83 (11%)**	
**BLOOD TEST**									
Very nervous	15/38 (39%)	5/9 (56%)	1/9 (11%)	3/9 (33%)	26/92 (28%)	15/23 (65%)	5/23 (22%)	3/23 (13%)	**41/130 (32%)**
A little bit nervous	9/38 (24%)	7/8 (87%)	1/8 (13%)	0/8 (0%)	32/92 (35%)	14/30 (47%)	13/30 (43%)	3/30 (10%)	**41/130 (32%)**
Don’t mind, not worried	5/38 (13%)	2/3 (67%)	1/3 (33%)	0/3 (0%)	19/92 (21%)	6/19 (32%)	13/19 (68%)	0/19 (0%)	**24/130 (18%)**
Looking forward to it	9/38 (24%)	3/5 (60%)	2/5 (40%)	0/5 (0%)	15/92 (16%)	8/12 (67%)	4/12 (33%)	0/12 (0%)	**24/130 (18%)**
		**17/25 (68%)**	**5/25 (20%)**	**3/25 (12%)**		**43/84 (51%)**	**35/84 (42%)**	**6/84 (7%)**	
**ORAL FLUID SAMPLE**									
Very nervous	4/37 (11%)	2/3 (67%)	1/3 (33%)	0/3 (0%)	6/95 (6%)	3/6 (50%)	2/6 (33%)	1/6 (17%)	**10/132 (8%)**
A little bit nervous	5/37 (13%)	1/3 (33%)	2/3 (67%)	0/3 (0%)	10/95 (11%)	5/10 (50%)	5/10 (50%)	0/10 (0%)	**15/132 (11%)**
Don’t mind, not worried	13/37 (35%)	6/10 (60%)	2/10 (20%)	2/10 (20%)	64/95 (67%)	19/58 (33%)	27/58 (46%)	12/58 (21%)	**77/132 (58%)**
Looking forward to it	15/37 (41%)	11/12 (92%)	1/12 (8%)	0/12 (0%)	15/95 (16%)	8/14 (57%)	6/14 (43%)	0/14 (0%)	**30/132 (23%)**
		**20/28 (71%)**	**6/28 (21%)**	**2/28 (7%)**		**35/88 (40%)**	**40/88 (45%)**	**13/88 (15%)**	

Denominators for each question defer because not all participants completed all questions.

Older children (8–11 yrs) were generally more relaxed about the tests compared to younger children (4–7 yrs) with the majority reporting they were “not worried” or “looking forward to” the nose swab (64/93 [69%] vs. 13/39 [33%]; P = 0.041) and throat swab (60/95 [63%] vs. 20/39 [51%]; P = 0.516). However, 8–11 year olds did report that they were nervous about the blood test, similar to 4–7 year olds (58/92 [63%] vs. 24/38 [63%]; P = 0.995).

Pre-testing apprehension (“very nervous”) was similar between both age categories for three of the four tests (nose: 7/39 [18%] vs. 10/93 [11%]; P = 0.329, blood: 15/38 [39%] vs. 26/92 [28%]; P = 0.375, oral fluid samples: 4/37 [11%] vs. 6/95 [6%]; P = 0.421), while younger children reported significantly greater apprehension about throat swabs than older children (throat (9/39 [23%) vs. 8/95 [8%]; P = 0.047) (**Table 1**).

#### After testing

After completing their tests, a higher proportion of 4–7 year-olds reported the tests to have been “better than expected” compared to 8–11 year olds, including nose swabs (20/32 [62%] vs. 41/82 [50%]; P = 0.52), throat swabs (15/31 [48%] vs. 35/83 [42%]; P = 0.71), oral fluid sampling (20/31 [64%] vs. 35/88 [40%]: P = 0.17) and blood tests (17/26 [65%] vs. 43/85 [51%]; P = 0.48), although these differences were not statistically significant (**Table 1**).

Most children reported that all tests were “better” or “as expected” (86–95%), with a minority reporting that it was “worse” (5–14%). Even those who said they were “very nervous” about the tests reported that it was “better” than they expected. For the blood test, 5/9 (56%) of 4–7 year olds and 15/23 (65%) of 8–11 year olds who were “very nervous” beforehand, reported it was “better” than they had expected (P = 0.25). In older children aged 8–11 years, those who reported they were “not worried” about the tests, were more likely to indicate that the tests were “as they expected”, compared to 4–7 year olds who were more likely to say the test was “better” than they expected (**Table 1**). Children aged 8–11 years were more likely to report the tests to be “as expected” than 4–7 year olds. Only 2/26 (8%) of 4–7 year-olds reported the blood test to be as expected compared to 35/85 (41%) of 8–11 year olds (P = 0.015) (**Table 1**). When asked if they would have the tests again after their investigations, the students were more likely to agree to additional testing for nose swabs (61/113 [54%]) or to oral fluid (60/114 [53%]) [p = 0.838], followed by throat swabs (57/113 [50%]) and blood tests (40/108 [37%]) [p = 0.045] (**Table 2**). Younger children were significantly more likely to decline a repeat throat swab, blood test and oral fluid compared to older children (**Table 2**).

**Table 2:** Children’s responses when asked if they would have each individual test again.

	4–7 year-olds	8–11 year-olds	p-value	All children
**Repeat Nose Swab**				
Maybe	7/32 (22%)	25/81 (31%)	0.40	32/113 (28%)
No	11/32 (34%)	9/81 (11%)	0.004	20/113 (18%)
Yes	14/32 (44%)	47/81 (58%)	0.17	61/113 (54%)
**Repeat Throat Swab**				
Maybe	8/32 (25%)	20/81 (25%)	0.97	28/113 (25%)
No	15/32 (47%)	13/81 (16%)	0.001	28/113 (25%)
Yes	9/32 (28%)	48/81 (59%)	0.003	57/113 (50%)
**Repeat Blood Test**				
Maybe	3/25 (12%)	29/83 (35%)	0.028	32/108 (30%)
No	16/25 (64%)	20/83 (24%)	<0.001	36/108 (33%)
Yes	6/25 (24%)	34/83 (41%)	0.12	40/108 (37%)
**Repeat Oral Fluid Samples**				
Maybe	7/31 (23%)	26/83 (31%)	0.36	33/114 (29%)
No	14/31 (45%)	7/83 (9%)	<0.001	21/114 (18%)
Yes	10/31 (32%)	50/83 (60%)	0.008	60/114 (53%)

Denominators for each question defer because not all participants completed all questions.

### Frequency of testing in

#### Swabbing schools

In primary schools participating in weekly nasal swabbing, most parents and staff selected a preference for weekly nose swabbing (2445/3868 [63%], & 1209/2251 [54%]), throat swabbing (1374/3754 [37%] and 948/2186 [43%]) and oral fluid sample (1734/3779 [46%] and 1003/2188 [46%]) (**Table 3**). With respect to blood testing, 31% (673/2164) of staff and 11% (395/3676) of parents indicated they would tolerate blood tests once a week, while 10% (222/473) of staff refused to have a blood test and 43% (1584/3676) of parents refused blood tests for their child.

**Table 3:** Responses of Staff and Parents of children in primary schools in relation to frequency of testing for each investigation in schools participating in (A) weekly swabbing and (B) blood sampling.

**A) WEEKLY SWABBING SCHOOLS**						
**NOSE SWAB**	**Parents**		**Staff**			**All**
At the beginning and end of each half-term	60/3863 (2%)		28/2251 (1%)			88/6114 (2%)
At the beginning and end of each term	34/3863 (1%)		11/2251 (0%)			45/6114 (1%)
Daily	341/3863 (9%)		277/2251 (12%)			618/6114 (10%)
I wouldn’t agree to have any more swabs done	41/3863 (1%)		16/2251 (1%)			57/6114 (1%)
Once a month	147/3863 (4%)		59/2251 (3%)			206/6114 (3%)
Once a week	2445/3863 (63%)		1209/2251 (54%)			3654/6114 (60%)
The frequency of swabbing does not bother me	666/3863 (17%)		570/2251 (25%)			1236/6114 (20%)
Twice a week	129/3863 (3%)		81/2251 (4%)			210/6114 (3%)
**THROAT**						
At the beginning and end of each half-term	145/3754 (4%)		73/2186 (3%)			218/5940 (4%)
At the beginning and end of each term	71/3754 (2%)		35/2186 (2%)			106/5940 (2%)
Daily	233/3754 (6%)		220/2186 (10%)			453/5940 (8%)
I wouldn’t agree to have any more swabs done	942/3754 (25%)		172/2186 (8%)			1114/5940 (19%)
Once a month	340/3754 (9%)		137/2186 (7%)			477/5940 (8%)
Once a week	1374/3754 (37%)		948/2186 (43%)			2322/5940 (39%)
The frequency of swabbing does not bother me	569/3754 (15%)		533/2186 (24%)			1102/5940 (19%)
Twice a week	80/3754 (2%)		68/2186 (3%)			148/5940 (2%)
**ORAL FLUID**						
At the beginning and end of each half-term	96/3779 (3%)		42/2188 (2%)			138/5967 (2%)
At the beginning and end of each term	47/3779 (1%)		18/2188 (1%)			65/5967 (1%)
Daily	385/3779 (10%)		281/2188 (13%)			666/5967 (11%)
I wouldn’t agree to have any more swabs done	464/3779 (12%)		71/2188 (3%)			535/5967 (9%)
Once a month	223/3779 (6%)		88/2188 (4%)			311/5967 (5%)
Once a week	1734/3779 (46%)		1003/2188 (46%)			2737/5967 (46%)
The frequency of swabbing does not bother me	721/3779 (19%)		604/2188 (28%)			1325/5967 (22%)
Twice a week	109/3779 (3%)		81/2188 (4%)			190/5967 (3%)
**BLOOD TESTS**						
At the beginning and end of each half-term	301/3676 (8%)		129/2164 (6%)			430/5840 (7%)
At the beginning and end of each term	271/3676 (7%)		79/2164 (4%)			350/5840 (6%)
Daily	71/3676 (2%)		132/2164 (6%)			203/5840 (3%)
I wouldn’t agree to have any more tests done	1584/3676 (43%)		222/2164 (10%)			1806/5840 (31%)
Once a month	697/3676 (19%)		410/2164 (19%)			1107/5840 (19%)
Once a week	395/3676 (11%)		673/2164 (31%)			1068/5840 (18%)
The frequency of swabbing does not bother me	336/3676 (9%)		473/2164 (22%)			809/5840 (14%)
Twice a week	21/3676 (1%)		46/2164 (2%)			67/5840 (1%)
**B) BLOOD SAMPLING SCHOOLS**						
**NOSE SWAB**		**Parents**		**Staff**	**All**	
At the beginning and end of each half-term		63/495 (13%)		65/455 (14%)	128/950 (13%)	
At the beginning and end of each term		66/495 (13%)		47/455 (10%)	113/950 (12%)	
Daily		34/495 (7%)		31/455 (7%)	65/950 (7%)	
I wouldn’t agree to have any more swabs done		29/495 (6%)		14/455 (3%)	43/950 (5%)	
Once a month		104/495 (21%)		52/455 (11%)	156/950 (16%)	
Once a week		93/495 (19%)		80/455 (18%)	173/950 (18%)	
The frequency of swabbing does not bother me		94/495 (19%)		158/455 (35%)	252/950 (27%)	
Twice a week		12/495 (2%)		8/455 (2%)	20/950 (2%)	
**THROAT**						
At the beginning and end of each half-term		65/493 (13%)		69/452 (15%)	134/945 (14%)	
At the beginning and end of each term		69/493 (14%)		44/452 (10%)	113/945 (12%)	
Daily		33/493 (7%)		29/452 (6%)	62/945 (7%)	
I wouldn’t agree to have any more swabs done		40/493 (8%)		15/452 (3%)	55/945 (6%)	
Once a month		106/493 (22%)		58/452 (13%)	164/945 (17%)	
Once a week		83/493 (17%)		70/452 (15%)	153/945 (16%)	
The frequency of swabbing does not bother me		88/493 (18%)		159/452 (35%)	247/945 (26%)	
Twice a week		9/493 (2%)		8/452 (2%)	17/945 (2%)	
**ORAL FLUID**						
At the beginning and end of each half-term		57/495 (12%)		65/455 (14%)	122/950 (13%)	
At the beginning and end of each term		62/495 (13%)		46/455 (10%)	108/950 (11%)	
Daily		40/495 (8%)		37/455 (8%)	77/950 (8%)	
I wouldn’t agree to have any more swabs done		23/495 (5%)		12/455 (3%)	35/950 (4%)	
Once a month		102/495 (21%)		52/455 (11%)	154/950 (16%)	
Once a week		91/495 (18%)		70/455 (15%)	161/950 (17%)	
The frequency of swabbing does not bother me		107/495 (22%)		163/455 (36%)	270/950 (28%)	
Twice a week		13/495 (3%)		10/455 (2%)	23/950 (2%)	
**BLOOD TESTS**						
At the beginning and end of each half-term		66/480 (14%)		77/451 (17%)	143/931 (15%)	
At the beginning and end of each term		110/480 (23%)		43/451 (10%)	153/931 (16%)	
Daily		13/480 (3%)		22/451 (5%)	35/931 (4%)	
I wouldn’t agree to have any more tests done		71/480 (15%)		15/451 (3%)	86/931 (9%)	
Once a month		133/480 (28%)		63/451 (14%)	196/931 (21%)	
Once a week		32/480 (7%)		69/451 (15%)	101/931 (11%)	
The frequency of swabbing does not bother me		53/480 (11%)		153/451 (34%)	206/931 (22%)	
Twice a week		2/480 (0%)		9/451 (2%)	11/931 (1%)	

Denominators for each question defer because not all participants completed all questions.

#### Blood sampling schools

In blood sampling schools, around a third of school staff reported that the frequency of all testing methods did not bother them (nose: 158/455 [35%], throat: 159/452 [35%], oral fluid swabs: 163/455 [36%], blood: 153/451 [34%]). Parents, however, expressed a frequency preference of once a month for the tests: blood tests (133/480 [28%]), nose swabs (104/495 [21%]), oral fluids (102/495[21%]), and throat swabs (106/493 [22%]) (**Table 3**).

#### Free text comments

There were 711 free-text comments included in the questionnaires, mainly from parents (n = 640, 90%) than staff (n = 71, 10%). Of these, 456 comments (424 [93%] parents, 32 [7%] staff) in swabbing only schools were regarding feasibility and acceptability of the types of testing. The free text comments were filtered to focus on feedback relevant to the research question, with “no comment”, or other unrelated content (e.g. name, date of birth) removed. Other comments that were not included in this analysis related mainly to providing additional demographic information, details about previous illnesses in the family or participants expressing that that had no further comments. The remaining free-text comments were coded by one of the study team members.

In the blood sampling schools, there were 62 free text comments, including 34 (32 [94%] parents, 2 [6%] staff) regarding acceptability and feasibility of testing in educational status. There were no differences in the content of comments between blood sampling and swabbing schools, with the exception of a higher proportion of positive comments (e.g. gratitude for testing opportunity) expressed at the blood sampling schools (5/62 [8%] vs. 6/465 [1%], P = 0.001).

Qualitative analysis of the free text comments by parents identified 135 were regarding blood tests, predominantly questioning the need for or declining blood tests. Similarly, comments relating to “throat swabs” (n = 47) and “nose swabs” (n = 37) were mainly related to parents agreeing to these two tests but declining a blood test for their child.

- *“I would like further information as to why blood tests might be required… Further information might change our view*.*”*–parent, swabbing school
- *“…If they were uncomfortable for X then I would need to consider this further*. *For any test that are painless I am happy for them to be done as often as needed…”*–parent, swabbing school
- *“I wouldn’t like my child to have a blood test”*–parent, blood testing school

Other common comments involved the welfare and wellbeing of their child (n = 77), requesting more information before participating (n = 53) and wanting to be present for the investigations (n = 41):

- *“Can a parent be present for the tests as X may be scared*?*”*–parent, blood sampling school
- *“Interested in a blood test/antibody test but would like to be present and need more information”–*parent swabbing school
- *“Blood test only if parent notified and present when blood is taken”–*parent, blood testing school

Free text comments from staff were generally positive about the prospect of testing and none expressed any concerns regarding the sampling:

- *“I would also be interested in having the antibodies test when a suitable one is available”*–staff, swabbing school
- *“thank you for the opportunity to have test”*–staff, swabbing school
- *“I am grateful for this initiative”*–staff, blood testing school

### Swabbing participation

**Fig 1:**
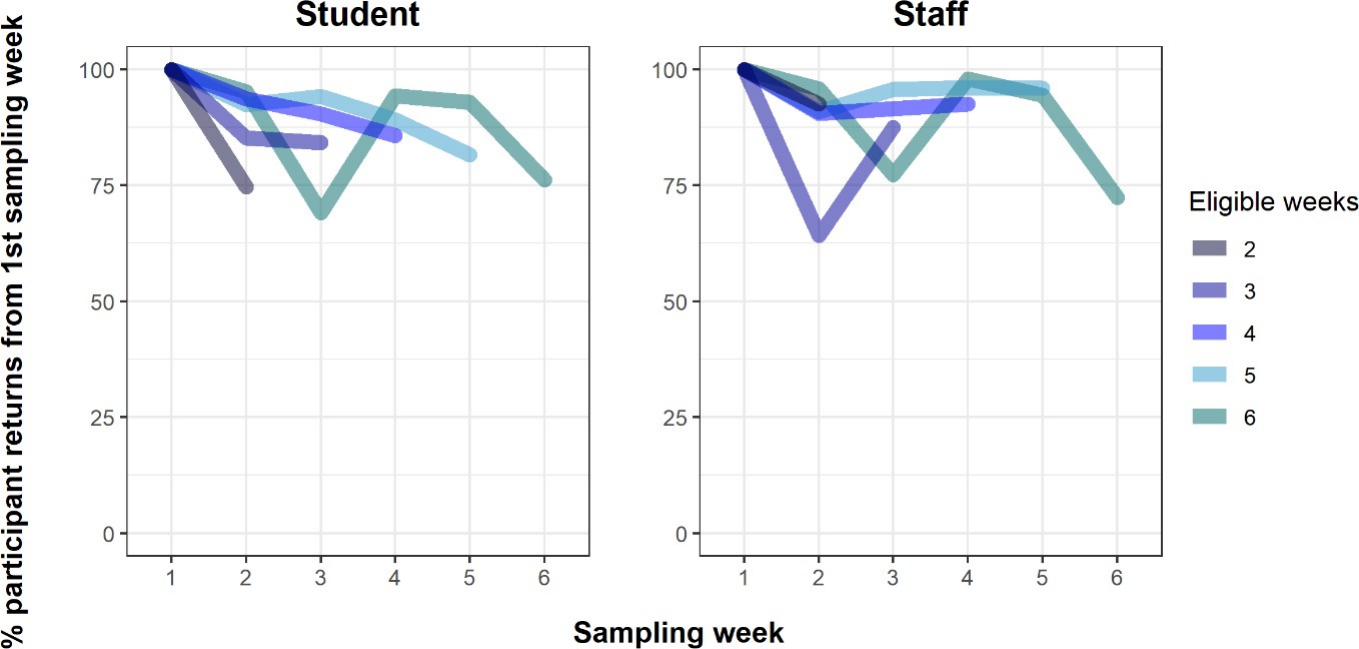
Participant return rates of students and staff per eligible sampling week.

Overall mean (±SD) return rates per week were 87.8% (8.98%), varying between 72.19% and 95.79% (recorded in the third and fourth sampling weeks for those eligible for six tests). These return rates were similar between students (86.6% ± 8.1%) and staff (89.0% ± 9.9%) and remained broadly consistent irrespective of eligible weeks (**Fig 1 and S1 Table**).

## Discussion

The COVID-19 pandemic had led to unprecedented changes in children’s lives. Most countries closed their schools for prolonged periods as part of national lockdown to control the spread of the virus [1]. School closure not only impacts the education of children, but also affects their personal, emotional and social growth, and limits access to free school meals and social services, thus disproportionately affecting the most disadvantaged families [13]. The reopening of schools after lockdown was, therefore, an important first step to establish normality for children, but has been highly divisive among educationalists, politicians, parents and policy makers [10].

Early in the pandemic, the role of children, particularly in relation to asymptomatic infection and transmission of SARS-CoV-2, was uncertain [14]. In England, therefore, schools only partially reopened in June 2020 and with strict social distancing and infection control practices in place [15]. Given concerns about widespread SARS-CoV-2 infection and transmission in educational settings, PHE initiated enhanced surveillance in primary schools with multiple objectives. These included nose and throat swabs to detect symptomatic and asymptomatic infections in staff and students, oral fluid samples to develop non-invasive SARS-CoV-2 antibody testing and blood sampling for SARS-CoV-2 antibodies to measure prior infection, even if asymptomatic or transient [11].

Whilst the surveillance continues to provide invaluable insights into SARS-CoV-2 infection and transmission in primary schools, our methodology provided a unique opportunity to assess the views of staff, parents and students on testing and surveillance educational settings. We found that the staff were willing to be tested more frequently compared to parents’ willingness for their children. In general, younger children were more nervous about the tests than older children but, after their investigations, most children reported a more positive experience with all the tests. Indeed, two-thirds of participating staff and students returned for at least one of their two subsequent blood sampling visits [11]. Additionally, when we explored participation rates for nasal swabbing across nearly 10,000 participants, we found very high weekly return rates (72–96%), illustrating that both students and staff were prepared to commit to regular testing.

### Comparisons to published literature

Parental attitudes and factors relating to paediatric venepuncture has previously been explored, but predominantly in hospital/clinical settings. Parental presence, support of healthcare staff and quality information prior to procedure have been associated with reduced stress and pain for the child, in addition to improving participation and collaboration between parents and healthcare staff [16, 17]. Similar themes were also identified in the qualitative analysis of parent comments in our analysis.

In England, schools are accustomed to public health interventions, such as the National Child Measurement Programme where children have their height and weight measured anuually [18], and receive some of their vaccines (e.g. HPV, MenACWY, nasal influenza) vaccinations [19]. Vaccination though, unlike venepuncture, has a clear personal benefit, which may account for the high uptake in England [19]. The sKIDs surveillance, however, was potentially more intrusive because it involved multiple investigations, including blood sampling which, although performed by experienced staff, was mainly performed in the absence of parents, even though they were aware of the investigations and had provided written consent. Furthermore, it relied on the good will of participants to improve our knowledge of SARS-CoV-2, with little or no personal benefit. Indeed, participation had a risk of testing positive for SARS-CoV-2, which would result in the child/bubble being sent home for isolation, thus further disrupting access to education and creating childcare issues for working parents.

Overall, however, the large numbers of participants is a testimony to their willingness to support research on SARS-CoV-2 infection so that schools could remain open safely during the pandemic. We observed a higher return rates for weekly nasal swabbing (88%) compared to blood sampling (62–65%) [11], which is not surprising, but this was important because serum SARS-CoV-2 antibodies provide more robust evidence of prior exposure to the virus, including transient and asymptomatic infections [20]. Contemporaneous collection of oral fluid samples also allowed the development and validation of a non-invasive antibody test, which is now being used in a larger School Infection Survey (SIS) aiming to recruit up to 50,000 staff and students in 150 schools across England [21]. The willingness of the children to take part in this important investigation and the high retention rate for subsequent visits despite their nervousness about all the tests is commendable.

In the published literature, there are limited studies on the feasibility and acceptability of mass testing in schools. A study from 1994 in England investigated the feasibility and acceptability of venepuncture in schools, measuring haemoglobin, ferritin and cholesterol in school children [22]. They found no significant difference in refusal rates between families of children who received an explanation about the test before entering the testing room and those who received an explanation at the time of testing. The study also found lower response rates from parents in low- compared to high-socioeconomic groups [22]. Interestingly, the authors reported that some parents asked to be present during the procedure, but this often exacerbated the stress and anxiety of the child, which is similar to our experience. Indeed, allowing children to participate in the surveillance without their parents could potentially be beneficial through promotion of self-confidence and independence.

### Limitations

The questionnaires were completed by staff and parents who had already agreed to take part in the sKIDs surveillance. This group may, therefore, not be representative of all staff and parents of children in primary schools. Similarly, the children completing the questionnaires had already agreed to take part and, since only six London schools were involved, may not be representative of all primary school children. We observed for example, that parental and staff preference for testing was influenced by the sKIDs investigations in those schools such that parents/staff in the blood sampling arm were more accepting of blood testing and frequent blood testing compared to those in the weekly swabbing schools. This is most likely explained by the fact that the parents in the blood sampling arm had already agreed for their children to have the blood test.

### Implications and conclusions

The sKIDs surveillance has contributed significantly to our understanding of SARS-CoV-2 infection and transmission in educational settings and helped support the decision to reopen all school years in September 2020. The scale of the sKIDS surveillance was unprecedented, given that these were undertaken in the middle of a pandemic and involved multiple investigations including blood sampling in young children. We found that high-level surveillance is both positively received by this demographic and that there is a clear willingness to participate. Involving the children in the surveillance also provided an opportunity to help improve health literacy, particularly in relation to the pandemic, and provided the children with a greater understanding of public health surveillance, thus facilitating interest and discussion in science.

## Conclusions

Taken together, our findings provide pervasive evidence that rapid surveillance in educational settings under pandemic conditions is achievable. We found that staff were willing to have more frequent testing than parents of primary school children. Also, whilst staff were less concerned about nature of the tests, nose swabs and oral fluids were more acceptable for children than throat swabs or blood sampling. The two-thirds return rate for repeat blood sampling in staff and children is a useful estimate for drop-out rates when estimating sample sizes for future large-scale blood testing in educational settings. Ongoing SARS-CoV-2 surveillance in educational settings during this pandemic remains key to keeping schools open safely. While our work concerns SARS-CoV-2 surveillance in primary school children, at its broadest level this work has significant implications for future surveillance programs in educational settings in this demographic.

## Supporting information

S1 File Feasibility questionnaire for staff and parents at recruitment (June 2020). (PDF)

S2 File Questionnaire for children in blood sampling arm. (PDF)

S1 Table Overall participant return rates by eligible sampling weeks. The first sample week per eligible sample week is 100%, with participation rate the number of returning individuals from this first week.
(PDF)

S1 Data (XLSX)
