# Supplementary material for: Feasibility and acceptability of SARS-CoV-2 testing and surveillance in primary school children in England: Prospective, cross-sectional study
Source: PLoS One. 2021 Aug 27;16(8):e0255517. doi: 10.1371/journal.pone.0255517 (PMC8396768; doi:10.1371/journal.pone.0255517)
Supplement: S1 File — (PDF) [file pone.0255517.s001.pdf]

Supporting Questionnaire (S1): Feasibility questionnaire for staff and parents at recruitment (June 2020)

FEASIBILITY QUESTIONS

We are trying to assess the practicalities of testing school staff more regularly

1. ***If you had a nose swab today, how frequently would you agree to have a nose swab in the future? (tick one)***
   - ☐ Daily
   - ☐ Twice a week
   - ☐ Once a week
   - ☐ Once a month
   - ☐ At the beginning and end of each half-term
   - ☐ At the beginning and end of each term
   - ☐ The frequency of swabbing does not bother me
   - ☐ I wouldn't agree to have any more swabs done

2. ***If you had a throat swab today, how frequently would you agree to have a throat swab in the future? (tick one)***
   - ☐ Daily
   - ☐ Twice a week
   - ☐ Once a week
   - ☐ Once a month
   - ☐ At the beginning and end of each half-term
   - ☐ At the beginning and end of each term
   - ☐ The frequency of swabbing does not bother me
   - ☐ I wouldn't agree to have any more swabs done

3. ***If you had a saliva swab today, how frequently would you agree to have a saliva swab in the future? (tick one)***
   - ☐ Daily
   - ☐ Twice a week
   - ☐ Once a week
   - ☐ Once a month
   - ☐ At the beginning and end of each half-term
   - ☐ At the beginning and end of each term
   - ☐ The frequency of swabbing does not bother me
   - ☐ I wouldn't agree to have any more swabs done

4. ***If you had a blood test today, how frequently would you agree to have a blood in the future? (tick one)***
   - ☐ Daily
   - ☐ Twice a week
   - ☐ Once a week
   - ☐ Once a month
   - ☐ At the beginning and end of each half-term
   - ☐ At the beginning and end of each term
   - ☐ The frequency of swabbing does not bother me
   - ☐ I wouldn't agree to have any more swabs done

5. ***How would you rate your overall experience today?***

   A. Very dissatisfied B. Dissatisfied C. Neutral D. Satisfied E. Very satisfied

6. ***Any further comments:***
