# Supplementary material for: Feasibility and acceptability of SARS-CoV-2 testing and surveillance in primary school children in England: Prospective, cross-sectional study
Source: PLoS One. 2021 Aug 27;16(8):e0255517. doi: 10.1371/journal.pone.0255517 (PMC8396768; doi:10.1371/journal.pone.0255517)
Supplement: S2 File — (PDF) [file pone.0255517.s002.pdf]

Supporting Questionnaire (S2): Questionnaire for children in blood sampling arm

COVID-19: sKIDS Study Questionnaire

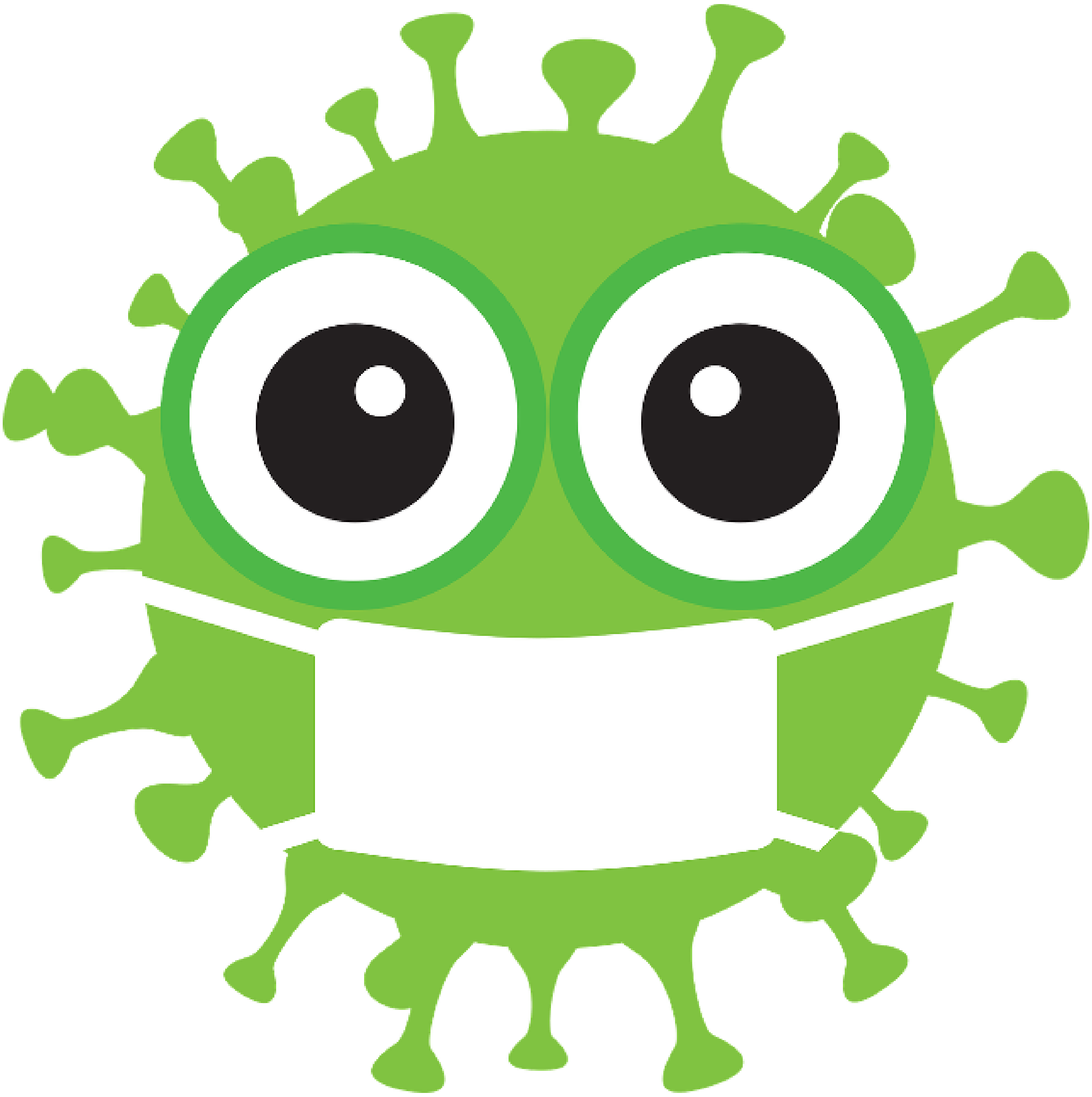

Participant Number:

We are going to take a nose and throat swab, blood test and saliva test to see if you have had the COVID-19 virus.

The nose swab looks like this:

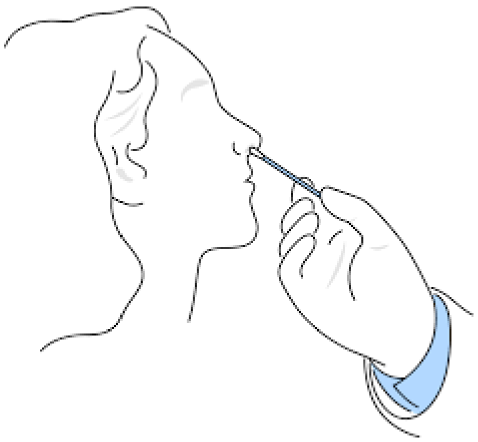

A small cotton bud which tickles the inside of both your nostrils. It might make you sneeze!

How are you feeling about your nose swab?

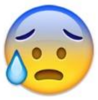

Very nervous!

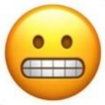

A little bit nervous

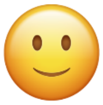

I don't mind, I'm not worried

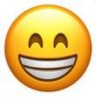

I'm looking forward to it!

The throat swab looks like this:

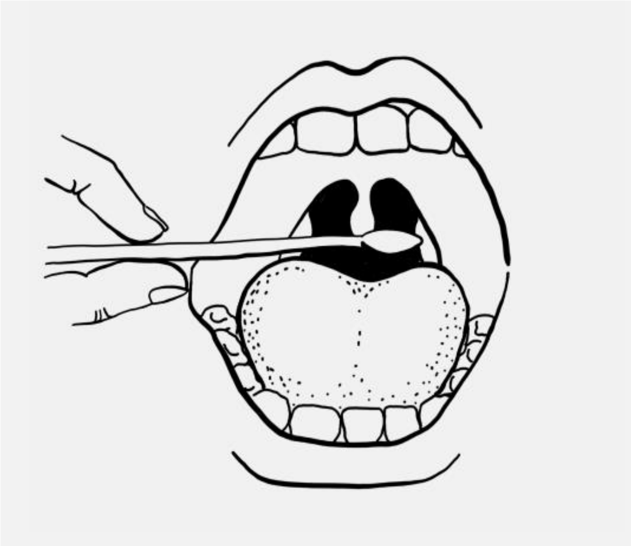

We will tickle both sides of the back of your throat with a small cotton bud. It might make you cough!

How are you feeling about your throat swab?

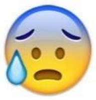

Very nervous!

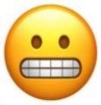

A little bit nervous

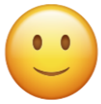

I don’t mind, I’m not worried

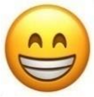

I’m looking forward to it!

The blood test looks like this:

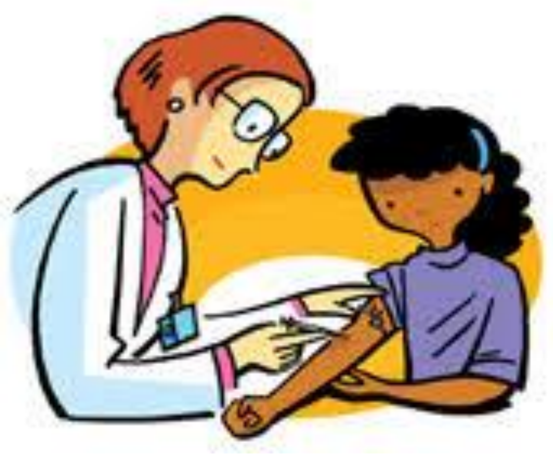

A tiny needle will take blood from your arm or the back of your hand. We will look for your best vein! We will also put on some special numbing cream so that it doesn't hurt.

We will take a tube of blood. We will also put some drops of blood on a card.

How are you feeling about your blood test?

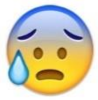

Very nervous!

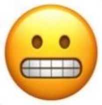

A little bit nervous

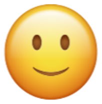

I don't mind, I'm not worried

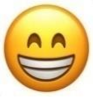

I'm looking forward to it!

The saliva test looks like this:

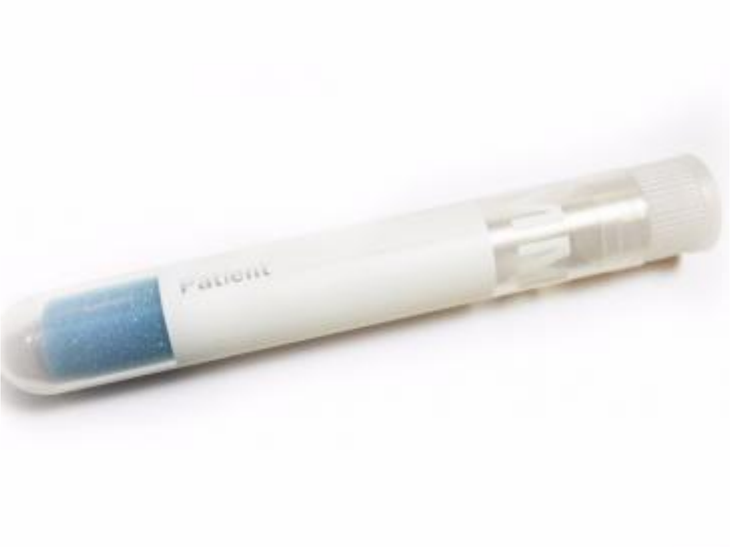

It is a blue sponge lollipop that you put in your mouth. Get it soggy with as much spit as possible! Wipe it around the inside of your cheeks and gums – a bit like when you brush your teeth.

How are you feeling about your saliva test?

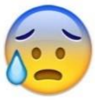

Very nervous!

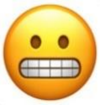

A little bit nervous

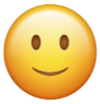

I don’t mind, I’m not worried

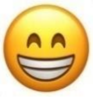

I’m looking forward to it!

How did you find the nose swab? Did it hurt?

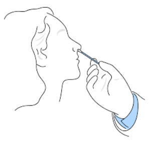

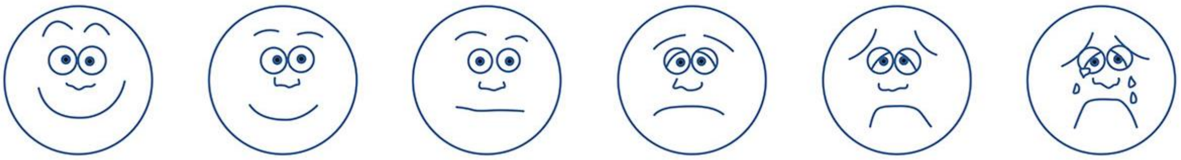

0	2	4	6	8	10
No Hurt	Hurts Little Bit	Hurts Little More	Hurts Even More	Hurts Whole Lot	Hurts Worst

Was it as you expected, better or worse?

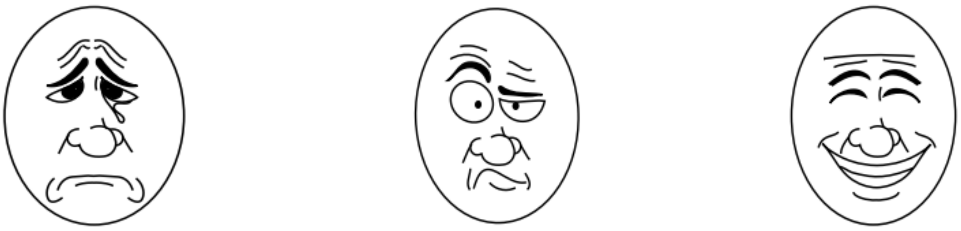

Worse than I thought

What I thought it would be like

Better than I thought it was going to be

Would you do it again?

Yes **No** **Maybe**

How did you find the throat swab? Did it hurt?

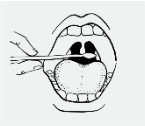

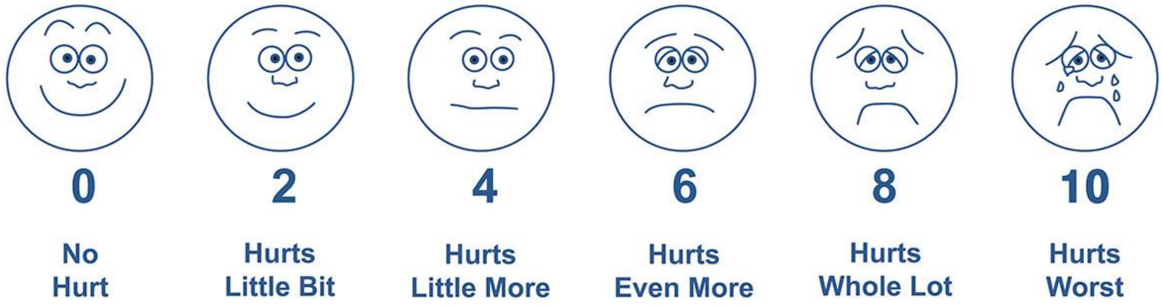

Was it as you expected, better or worse?

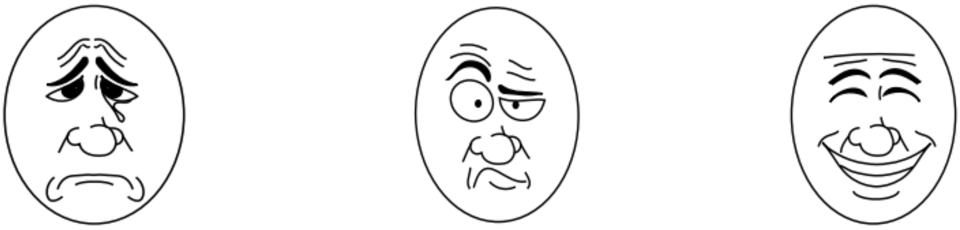

Worse than I thought

What I thought it would be like

Better than I thought it was going to be

Would you do it again?

Yes **No** **Maybe**

How did you find the blood test? Did it hurt?

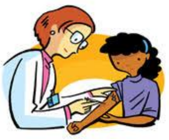

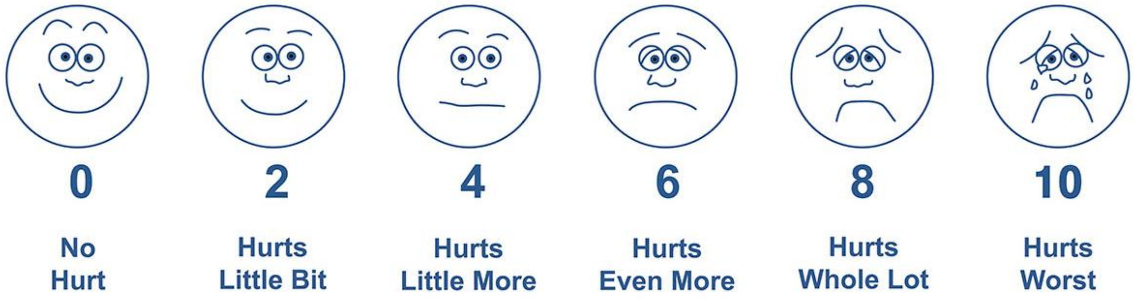

Was it as you expected, better or worse?

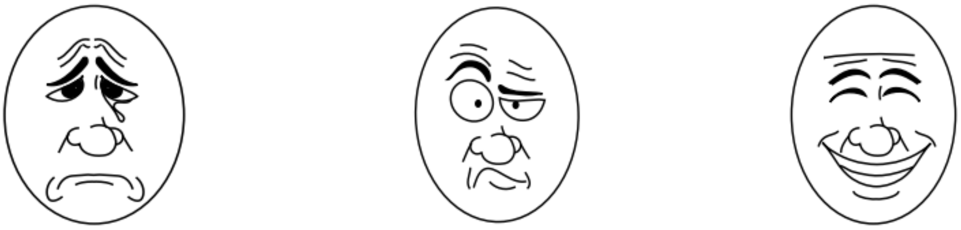

Worse than I thought	What I thought it would be like	Better than I thought it was going to be

Would you do it again?

Yes **No** **Maybe**

How did you find the saliva test? 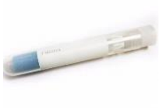

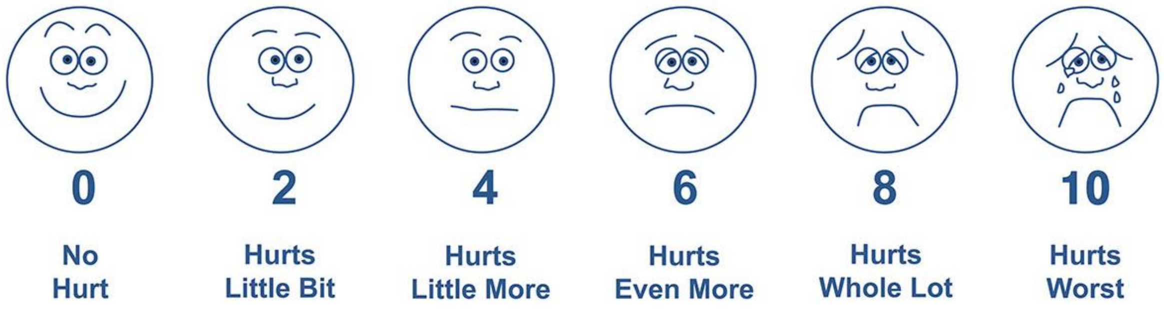

Was it as you expected, better or worse?

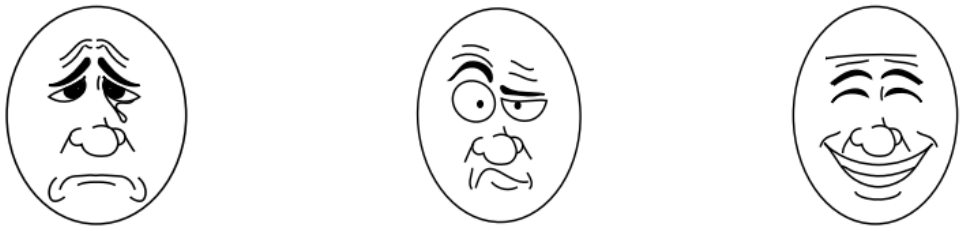

Worse than I thought	What I thought it would be like	Better than I thought it was going to be

Would you do it again?

Yes	No	Maybe

If you had to rank which test you liked the best to which you liked the least, what order would you put the tests in?

Where 1 = best, 4 = worst. Write your numbers in the boxes below each test.

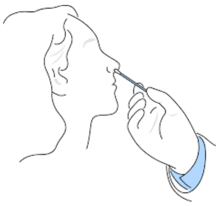

Nose swab

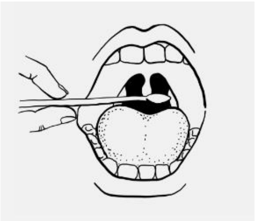

Throat swab

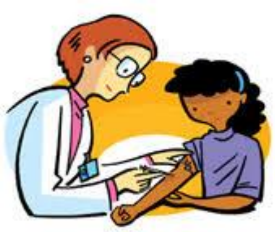

Blood test

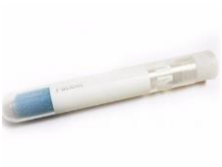

Saliva test

Thank you!
