# Supplementary material for: Feasibility and acceptability of SARS-CoV-2 testing and surveillance in primary school children in England: Prospective, cross-sectional study
Source: PLoS One. 2021 Aug 27;16(8):e0255517. doi: 10.1371/journal.pone.0255517 (PMC8396768; doi:10.1371/journal.pone.0255517)
Supplement: S1 Table — The first sample week per eligible sample week is 100%, with participation rate the number of returning individuals from this first week. (PDF) [file pone.0255517.s003.pdf]

S1 Table: Overall participant return rates by eligible sampling weeks. The first sample week per eligible sample week is 100%, with participation rate the number of returning individuals from this first week (linked to Figure 1).

Eligible sampling weeks	Sample week	Participants	% returns
2	1	520	100.00
	2	399	76.73
3	1	1003	100.00
	2	805	80.26
	3	838	83.55
4	1	4014	100.00
	2	3688	91.88
	3	3622	90.23
	4	3497	87.12
5	1	3418	100.00
	2	3134	91.69
	3	3223	94.29
	4	3121	91.31
	5	2968	86.83
6	1	356	100.00
	2	340	95.51
	3	257	72.19
	4	341	95.79
	5	333	93.54
	6	265	74.44
